# Effect of Previous Chemotherapy on the Quality of Cryopreserved Human Ovarian Tissue *In Vitro*

**DOI:** 10.1371/journal.pone.0133985

**Published:** 2015-07-30

**Authors:** Babak Asadi Azarbaijani, Mona Sheikhi, Irma C. Oskam, Mirja Nurmio, Tiina Laine, Helena Tinkanen, Sirpa Mäkinen, Tom G. Tanbo, Outi Hovatta, Kirsi Jahnukainen

**Affiliations:** 1 Women and Children’s Division, Oslo University Hospital, Rikshospitalet, Oslo, Norway; 2 University of Oslo, Oslo, Norway; 3 Division of Obstetrics and Gynecology, Karolinska Institute, Karolinska University Hospital, Huddinge, Stockholm, Sweden; 4 Stockholm IVF, Stockholm, Sweden; 5 Faculty of Veterinary Medicine and Bioscience, University of Oslo, Oslo, Norway; 6 Department of Physiology and Pediatrics, University of Turku, Turku, Finland; 7 Department of Pediatrics, Helsinki University Central Hospital, Helsinki, Finland; 8 Division of Obstetrics and Gynecology, University of Helsinki, Helsinki, Finland; 9 Tampere University Central Hospital, Tampere, Finland; 10 The Family Federation of Finland, Fertility Clinic, Helsinki, Finland; 11 Department of Women’s and Children´s Health, Karolinska Institute and University Hospital, Stockholm, Sweden; China Agricultural University, CHINA

## Abstract

**Background:**

Cryopreservation of ovarian tissue has been widely accepted as an option for fertility preservation among cancer patients. Some patients are exposed to chemotherapy prior to ovarian tissue cryopreservation. Consequently, assessment of the developmental capacity of human ovarian tissue after chemotherapy is of primary importance.

**Materials:**

In order to study the impact of previous chemotherapy on *in vitro* development and viability of ovarian follicles, quality control samples from 34 female cancer patients at median age of 15 years (range 1‒35), cryopreserved for fertility preservation before (n = 14) or after (n = 20) initiation of chemotherapy, were thawed and cultured for 7 days. The morphology and developmental stages of ovarian follicles were studied by light microscopy before and after culture. Possible associations between follicular densities, age and exposure to alkylating agents, expressed as cyclophosphamide equivalent dose (CED) were tested.

**Results:**

Exposure to chemotherapy significantly impaired the survival and development of ovarian follicles in culture. After seven days, significantly higher densities of intermediary, primary and secondary follicles and lower densities of atretic follicles was detected in the samples collected before chemotherapy. Increasing dose of alkylating agents was identified by multivariate linear regression analysis as an independent predictor of a higher density of atretic follicles, whereas increasing age of the patient predicted a better outcome with less follicle atresia and a higher density of maturing follicles.

**Conclusion:**

This study provides quantitative *in vitro* evidence of the impact of chemotherapy on developmental capacity of cryopreserved human ovarian tissue. The results indicate that fertility preservation should be carried out, if possible, before initiation of alkylating agents in order to guarantee better *in vitro* survival of ovarian follicles. In addition, ovarian samples from younger girls show lower viability and fewer developing follicles in culture.

## Introduction

Infertility is one of the late effects of cancer treatment among female survivors. Cryopreservation of ovarian tissue has been widely accepted as an option for fertility preservation [1]. It is the only option available for pre-pubertal girls and women who cannot delay the start of chemotherapy [1;2]. To achieve fertility, the follicles in the cryopreserved ovarian tissue ought to undergo full maturation from primordial follicles to antral follicles containing fully mature oocytes. At present, only patients with cancers associated with a low risk of ovarian metastasis are considered for auto-transplantation of ovarian tissue [3–5]. For the time being, it is not regarded safe to perform auto-transplantation on patients with high risk of ovarian metastasis, such as those with hematological cancers, because of possibility to reseeding malignant cells into cured patients [3–5].

Maturation of ovarian follicles and oocytes *in vitro* is a promising but challenging strategy to overcome the problems of cancer contamination. Even though there is progress in the procedures [6–9], full maturity with fertilizable oocytes has so far not been feasible in humans. In experimental animals, such as rodents, the procedure is shorter. The first live mouse was born from *in vitro* matured oocytes in 1996 [10], followed by large numbers of healthy offspring after merely improving the culture conditions [11]. New culture methods with using inhibitor of phosphatase and tensin homologue (PTEN) have been successfully used in activation of the primordial follicles *in vitro* and generation of fertilized egg in mouse [12]. Now, after the encouraging data on the mouse oocyte maturation, the *in vitro* methods need to be refined so that they can be applied to human follicles. We and others have successfully used PTEN inhibitor in cultures of human ovarian follicles during the first day of culture to promote follicle activation and development to the secondary stage [13;14].

The sterilizing effect of chemotherapy on the ovary is well known [15;16] and most oncologists recommend cryopreservation of ovarian tissue before initiation of chemotherapy [3]. Childhood cancers and hematological malignancies require prompt initiation of cancer therapy and the patients are therefore often exposed to chemotherapy prior to ovarian biopsy [17–19]. Up to date, there is no data regarding the effect of chemotherapy on *in vitro* development and survival of ovarian follicles in cryopreserved human ovarian tissue, despite its high importance for the feasibility of obtaining oocytes from the cryopreserved tissue.

In the present study, we used *in vitro* tissue culture to evaluate developmental capacity of cryopreserved human ovarian tissue taken as quality control samples for fertility preservation of children and young women with cancer. The study was conducted with special emphasis on the effects of chemotherapy with alkylating agents on the *in vitro* development and viability of ovarian follicles.

## Materials and Methods

### Tissue donors

Patients for fertility preservation were recruited at the Children's Hospital, University Central Hospital of Helsinki, Finland, the Department of Gynecology, Oslo University Hospital, Norway and the Department of Gynecology, University Hospital of Tampere, Finland [17].

Adult patients in the Oslo University Hospital and the University Hospital of Tampere were offered cryopreservation of ovarian tissue as a part of the fertility preservation program, which provided them with a full range of fertility-saving options. The participants signed an informed consent form for quality control of ovarian tissue including morphological analysis and *in vitro* culture. Ethical approval was therefore not necessary. All samples were anonymized before access and start of analysis.

In the Children's Hospital, Helsinki fertility preservation was performed as a part of research protocol approved by the Ethics Committee of Helsinki University Central Hospital. All age-appropriate patients or guardians provided their written informed consent for participation in the study.

Parental written consent was obtained from all patients under 18 years old.

Ovarian tissues used for control of the culture method were donated by four healthy women undergoing Cesarean section at Karolinska University Hospital, Stockholm, Sweden. They had signed an informed consent form for donation of a small piece of tissue for this research. The study and the consent form had been approved by The Regional Ethics Board in Stockholm.

The study samples were consecutive quality-control material for fertility preservation from patients with hematological and solid cancers (Helsinki, n = 19; Oslo, n = 13; Tampere n = 2) (Table 1) at median age of 15 years (range 1–35) at the time of biopsy. It was not possible to select tissue donors on the basis of age. Of the total of 34 samples obtained, 14 were collected at the time of diagnosis before chemotherapy and 20 after initiation of chemotherapy. The median age of healthy controls was 33 years (range 31–36).

**Table 1:** Controls and cancer patients who underwent biopsy before or after chemotherapy.

ID	Age (y)	Cryo-protectant	Cancer diagnosis	CED^a^ (mg/m^2^)	Interval form last chemotherapy to ovarian biopsy (day)
**Healthy donors**					
1	32	PrOH	-	-	-
2	36	PrOH	-	-	-
3	33	PrOH	-	-	-
4	31	PrOH	-	-	-
**Patients biopsied before chemotherapy**					
5	15	PrOH	Neuroblastoma	-	-
6	12	EG	Ewing Sarcoma	-	-
7	24	EG	Ewing Sarcoma	-	-
8	16	EG	Osteosarcoma	-	-
9	19	PrOH	Non-Hodgkin Lymphoma	-	-
10	20	EG	Hodgkin Lymphoma	-	-
11	23	EG	Hodgkin Lymphoma	-	-
12	14	EG	Hodgkin Lymphoma	-	-
13	22	PrOH	Acute Lymphocytic Leukemia	-	-
14	15	PrOH	Burkitt’s Lymphoma	-	-
15	35	PrOH	Acute Myeloid Leukemia	-	-
16	15	PrOH	Aplastic Anemia	-	-
17	19	PrOH	Acute Lymphocytic Leukemia	-	-
18	19	PrOH	Acute Lymphocytic Leukemia	-	-
**Patients biopsied after chemotherapy**					
19	3	PrOH	Neuroblastoma	7000	14
20	1	PrOH	Neuroblastoma	16640	55
21	2	PrOH	Neuroblastoma	7200	30
22	12	PrOH	Ewing Sarcoma	31560	21
23	10	EG	Rhabdomyosarcoma	8540	14
24	15	PrOH	Non-Hodgkin Lymphoma	4100	18
25	20	PrOH	Non-Hodgkin Lymphoma	6200	35
26	1	PrOH	Acute Lymphocytic Leukemia	2000	28
27	9	EG	Burkitt’s Lymphoma	4300	20
28	11	PrOH	Acute Lymphocytic Leukemia	6000	21
29	16	PrOH	Acute Lymphocytic Leukemia	2000	18
30	5	PrOH	Acute Lymphocytic Leukemia	3600	11
31	5	PrOH	Acute Lymphocytic Leukemia	4000	17
32	13	PrOH	Acute Lymphocytic Leukemia	4684	30
33^b^	6	PrOH	Acute Myeloid Leukemia	0	30
34	15	PrOH	Acute Lymphocytic Leukemia	7300	9
35	7	PrOH	Acute Lymphocytic Leukemia	4400	50
36	24	EG	Acute Lymphocytic Leukemia	4800	21
37	8	PrOH	Acute Lymphocytic Leukemia	6000	30
38	5	PrOH	Rhabdomyosarcoma	10248	17

PrOH = propanediol, EG = ethylene glycol

^a^Exposure to alkylating agents is indicated by cumulative Cyclophosphamide Equivalent Dose (CED).

^b^Treated with non-alkylating agents,

The hospitals’ medical records were used to collect information regarding the patients; i.e. age, diagnosis, type of treatment, timing and cumulative doses of chemotherapeutic agents. Cumulative exposure to alkylating agents was assessed by calculating the cyclophosphamide equivalent dose (CED), as described before [20]. Except for one, all patients treated with chemotherapeutic drugs received alkylating agents, cyclophosphamide being a major treatment element (Table 1). The interval between last dose of chemotherapy and ovarian biopsy was less than 56 days for all patients treated with chemotherapy (Table 1).

### Ovarian tissue cryopreservation and thawing

Ovarian tissue was biopsied by laparotomy or laparoscopy and transported on ice to the laboratory in cold phosphate-buffered saline prior to cryopreservation. Ovarian tissue from each patient was prepared in the laboratory by cutting the ovarian cortex into small pieces. Cryopreservation of ovarian tissue was carried out by using a slow freezing method with either propanediol (PrOH) or ethylene glycol (EG) as cryoprotectant agent depending on the cryopreservation protocol used in the respective hospital. The protocols for freezing of human ovarian tissue have been described earlier [21;22]. A programmable freezer was used and the tissue specimens were stored in liquid nitrogen. The cryopreserved pieces were thawed according to the reverse cryopreservation procedure [21;23;24]. The procedure took place at room temperature under a laminar flow hood in sterile conditions before transfer to culture medium.

### Ovarian tissue culture

Ovarian tissue culture was based on an established method as previously described [6;13;25;26]. The thawed ovarian cortical tissue was cut into pieces of 2–3 × 3–4 × 1.5 mm^3^. One piece was fixed in Bouin’s solution for histology. The remaining pieces were immediately cultured at 37°C in a humidified atmosphere containing 5% CO_2_, in 0.5 ml Dulbecco’s Modified Eagle’s Medium with Glutamax (Gibco, Invitrogen Inc.) supplemented with human serum albumin (10%; Vitrolife, Goteborg, Sweden), glutamine (3mM), follicle-stimulating hormone (0.5IU/ml; Gonal-F Serono Nordic Inc.), insulin-transferrin-selenium (1%; Invitrogen Inc.) and antibiotic/antimycotic (50IU/ml; Invitrogen Inc.). All samples were treated with phosphatase and tensin inhibitor (1 μM; Calbiochem, Merck Chemicals Ltd.) for the first 24 hours of the culture period to activate the growth of ovarian follicles. The culture medium was changed daily. The cultured ovarian tissue pieces were fixed in Bouin’s solution after seven days.

### Histology

The fixed ovarian tissue samples were placed in 70% alcohol after 24 h and stored at 4°C. To analyze the development of ovarian follicles by light microscopy, the dehydrated ovarian tissue strips were embedded in paraffin, sectioned (4 μm), and stained with hematoxylin and eosin (HE). Two persons counted the number of follicles at each developmental stage in all sections and controlled the results by use of an inter-observer variation method. To avoid double counting, each follicle was followed through neighboring sections.

**Fig 1:**
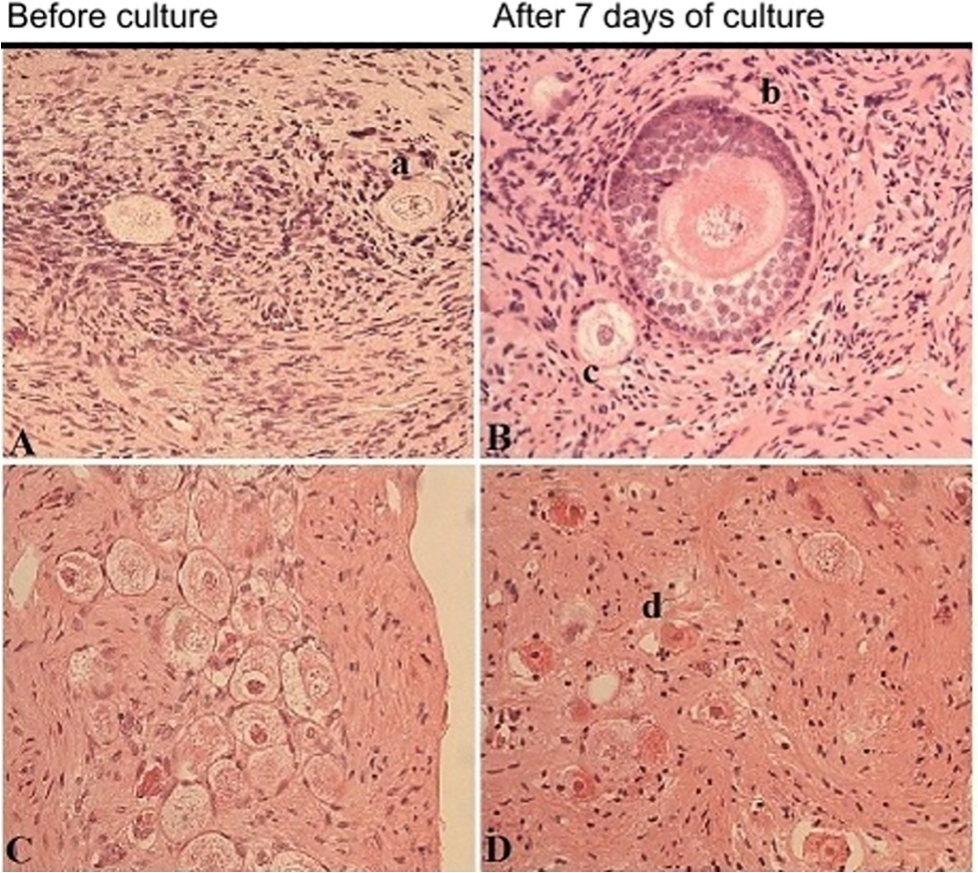
Representative images of ovarian cortex before and after seven days of culture (magnification ×40), from a 15-year-old girl with lymphoma and no chemotherapy (A,B), and from a 2-year-old girl with neuroblastoma exposed to CED of 7200 mg/m^2^ (C,D). a) Intact primordial follicle, b) intact secondary follicle, c) influenced primordial follicle, d) atretic follicle.

Follicles were classified as primordial, intermediary, primary and secondary (Fig 1) [26;27]. They were further classified as intact, influenced and atretic in order to evaluate the quality of the ovarian cortical pieces (Fig 1). Intact follicles were defined as those with an intact basement membrane attached to granulosa cells and without contraction of the cytoplasm or any pyknotic nuclei. The oocytes were in close contact with the surrounding granulosa cells, and the granulosa cells were without pyknotic nuclei or any signs of shrinkage or swelling. Influenced follicles were defined as those having intact nuclei and membranes of the oocyte, with less than 50% detachment of the oocyte from surrounding granulosa cells and/or less than 10% vacuolization in cytoplasm, and less than 50% atretic granulosa cells. The follicles were defined as atretic if the nucleus or more than 50% of any of the follicle structures described above were pyknotic. All intact and influenced primordial, intermediary, primary and secondary follicles were evaluated separately in each category.

The volume (V) of ovarian cortices was calculated by summing the number (n) of sections, of area Amm^2^ and thickness 0.004 mm: *V* (mm^3^) = (*A1*+*A2*+…+*An*) × 0.004. The areas were measured by using Nikon's NIS-Elements with ×4 magnification. The densities of each type of follicle in the ovarian cortex were then calculated as the total number of follicles divided by the total volume and expressed as the number of follicles/mm^3^ of ovarian tissue [28].

### Statistical analysis

SPSS statistical software version 21 was used to analyze the data. All data are presented as median and interquartile range (IQR). The Mann–Whitney *U* test was used to compare follicle densities in tissue samples removed before chemotherapy *vs*. those removed after initiation of chemotherapy. The entire study material was included in Spearman’s rank correlation analysis to assess univariate correlations between follicular density and age, and follicular density and CED. Follicular densities of the entire study material were further entered as dependent variables, and age, CED and cryoprotectant agents (PrOH or EG) as independent variables in multiple linear regression analysis. All tests of significance were two-tailed and p-values ≤ 0.05 indicated statistical significance.

## Results

The viability and developmental stages of a total of 17,212 non-cultured follicles and 41,231 follicles after seven days of culture were analyzed in tissues from 34 cancer patients and four healthy donors. Of a total of 34 samples, 33 were evaluated after thawing and 34 after seven days of culture. The results are based on ovarian follicular density (Table 2). Developmental stages from primordial to secondary follicles were observed after culture (Fig 1).

**Table 2:** Median and interquartile range (IQR) of follicle densities in cryopreserved human ovarian tissue before and after seven days of culture.

	No chemotherapy n = 14 Median (IQR)	Chemotherapy n = 20 Median (IQR)	P-value
Age (y)	19.0 (7.3)	8.5 (9.5)	**0.000**
CED^a^ (mg/m^2^)	0.0	5400 (3250)	**0.000**
**Follicle density before culture (per mm** ^**3**^ **)**			
Total follicles	155 (179)	263 (569)	0.194
Intact	103 (110)	85 (148)	0.434
Influenced	5 (37)	26 (75)	0.128
Atretic	0 (27)	74 (305)	**0.004**
Primordial	76 (86)	98 (183)	0.957
Intermediary	20 (41)	23 (43)	0.372
Primary	15 (11)	4 (9)	**0.005**
Secondary	0 (2)	0 (0)	0.785
**Follicle density after 7 days of culture (per mm** ^**3**^ **)**			
Total follicles	67 (103)	297 (417)	**0.027**
Intact	11 (34)	2 (9)	**0.011**
Influenced	7 (32)	6 (16)	0.500
Atretic	42 (64)	291 (407)	**0.004**
Primordial	9 (19)	5 (14)	0.192
Intermediary	8 (16)	2 (6)	**0.023**
Primary	7 (18)	0 (2)	**0.001**
Secondary	1 (3)	0 (0)	**0.000**

^a^ Cumulative Cyclophosphamide Equivalent Dose.

### Viability and development of ovarian follicles in control tissue

The samples from the four healthy donors were evaluated independently as controls for the culture method. Median density (IQR) of total, intact and atretic follicles were before culture 20 (31), 20 (31), 17 (10) and after culture 23 (17), 11 (5), 7 (8) per mm^3^, respectively. Median density of primordial, intermediary, primary and secondary follicles were before culture 6 (21), 6 (11), 6 (2), 0 (4) and after culture 4 (4), 4 (7), 6 (6), 1 (2) per mm^3^, respectively. The proportion of atretic follicles increased from 0% to 30% (P = 0.014) while the proportion of intact follicles decreased from 100% to 47% (P = 0.021) after the seven days of culture. No changes in the proportion of more developing follicles-primary and secondary follicles- were observed in culture (35% before and 40% after seven days of culture).

### Viability and development of follicles in ovarian tissue collected before and after the initiation of chemotherapy

Median follicular densities before and after culture are shown in Table 2. Before culture, the density of primordial follicles was similar in ovarian samples collected before and after the initiation of chemotherapy, however, more atretic follicles and fewer follicles in primary or secondary stages were seen in samples exposed to chemotherapy (Table 2). After seven days of culture the majority of follicles in samples collected after the initiation of chemotherapy had entered atresia (Table 2). Significantly higher densities of intermediary, primary and secondary follicles were detected in the samples collected before chemotherapy (Table 2).

The proportion of atretic follicles increased in culture. This was observed both in the samples not exposed (8% before and 53% after seven days of culture, P = 0.004) and exposed to chemotherapy (before 41%, after 86%, P = 0.070). The decrease in the proportion of intact follicles was more notable in samples exposed to chemotherapy (before 46%, after 6%, P<0.001) than in samples that were not exposed (before 82%, after 28%, P = 0.001). No changes in the proportions of more developing follicles-primary and secondary follicles- were detected in the samples that were not exposed to chemotherapy (before 17%, after 17%), while proportions of more developing follicles were decreased in the samples collected after exposure to chemotherapy (before 8%, after 5%, P = 0.014).

### Effects of age and ovarian exposure to alkylating agents on viability and development of ovarian follicles

**Fig 2:**
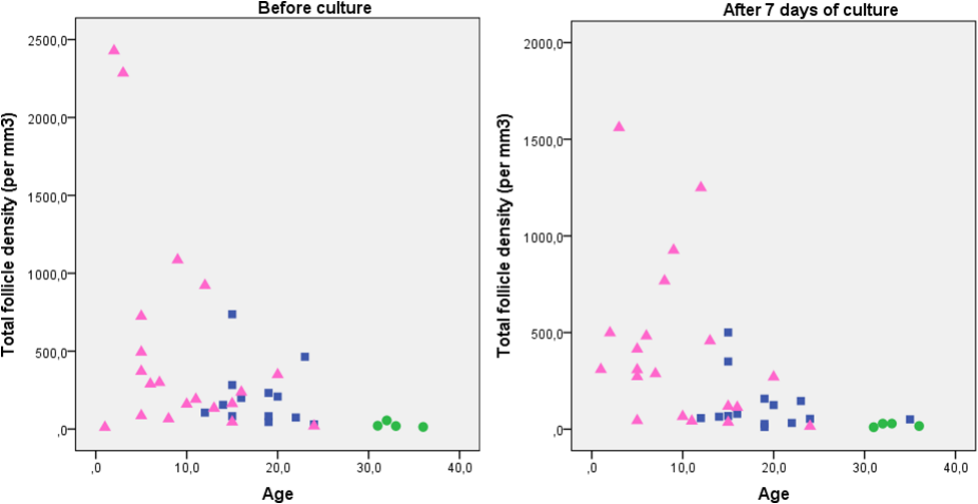
Relationship between follicle density and age before and after seven days of culture in controls (circles) and cancer patients with (triangles) and without (squares) chemotherapy.

Patients who had not received chemotherapy before ovarian biopsy were significantly older than those who had received chemotherapy (Tables 1 and 2, Fig 2). Exposure to alkylating agents, expressed as CED, varied significantly between the patients (Tables 1 and 2). Spearman’s rank correlation analysis was performed to identify if age or CED correlated to follicle densities using 33 samples before and 34 samples after seven days of culture (Table 3). Increasing age correlated significantly with an increasing density of developing follicles and with a lower density of total and atretic follicles both before and after seven days of culture (Table 3). Increasing exposure to CED correlated with an increased density and proportion of atretic follicles before and after culture (Table 3). Increased CED also correlated with a decreased density of developing follicles after culture (Table 3).

**Table 3:** Spearman’s rank correlation analysis of age and cumulative Cyclophosphamide Equivalent Dose (CED) and their association with follicle densities after seven days of culture.

	Age		CED	
	*Rho*	P-value	*Rho*	P-value
**Before culture**				
Total follicles	-0.362	**0.038**	0.342	0.052
Intact follicles	-0.050	0.784	0.029	0.873
Influenced follicles	-0.275	0.121	0.367	**0.036**
Atretic follicles	-0.532	**0.001**	0.474	**0.005**
Primordial follicles	-0.156	0.386	0.198	0.269
Intermediary follicles	0.345	**0.049**	-0.328	0.062
Primary follicles	0.060	0.739	-0.016	0.930
Secondary follicles	-0.103	0.567	-0.098	0.588
**After 7 days of culture**				
Total follicles	-0.563	**0.001**	0.345	**0.046**
Intact follicles	0.409	**0.016**	-0.281	0.108
Influenced follicles	0.046	0.796	-0.073	0.681
Atretic follicles	-0.646	**0.000**	0.413	**0.015**
Primordial follicles	0.096	0.591	-0.157	0.376
Intermediary follicles	0.590	**0.000**	-0.439	**0.009**
Primary follicles	0.351	**0.042**	-0.250	0.154
Secondary follicles	0.592	**0.000**	-0.519	**0.002**

*Rho* = Spearman's rank correlation coefficient

Multivariate linear regressions were performed to adjust for differences in age, cryoprotectant agents (PrOH or EG) and exposure to CED between the groups. Multivariate linear regression analysis identified increasing age of the patient as the only independent predictor of lower total density of follicles in ovarian samples before culture (Table 4). After culture for seven days, both age and exposure to alkylating agents independently predicted viability of ovarian follicles. Increasing age predicted a lower density of total and atretic follicles, while exposure to increasing CED predicted higher density of total and atretic follicles. Increasing age was the only independent predictor of higher density of the differentiating primary and secondary follicles after seven days of culture (Table 4). The cryoprotectants PrOH or EG were not significantly associated with follicle densities when adjusted to other factors in the regression model (Table 4).

**Table 4:** Multivariate linear regression analysis of follicle densities using age, cumulative Cyclophosphamide Equivalent Dose (CED) and propanediol (PrOH) as predictors after 7 days of culture.

Outcome variable	Predictor	B	SE_B_	P-value
**Before culture**				
Total follicles	Age	-32.62	15.80	**0.046**
R^2^ adj = 20%	CED	0.02	0.02	0.213
	PrOH	-11.59	259.30	0.965
**After 7 days culture**				
Total follicles	Age	-15.23	7.24	**0.044**
R^2^ adj = 22%	CED	0.02	0.01	**0.013**
	PrOH	106,55	220.47	0.632
Atretic follicles	Age	-15.81	7.00	**0.031**
R^2^ adj = 24%	CED	0.03	0.01	**0.006**
	PrOH	82,14	212.20	0.701
Primary follicles	Age	0.66	0.22	**0.004**
R^2^ adj = 12%	CED	0.00	0.00	0.473
	PrOH	-0.83	6.57	0.900
Secondary follicles	Age	0.14	0.03	**0.000**
R^2^ adj = 17%	CED	-0.00	0.00	0.590
	PrOH	-0.33	0.953	0.733

B = Regression coefficient, SE_B_ = Standard Error, R^2^ adj = Adjusted R-squared

Only significant differences are indicated.

## Discussion

The aim of ovarian tissue cryopreservation is to increase the chance of fertility in cured cancer patients, either via transplantation or *in vitro* culture. To achieve the goal, the evaluation of the developmental capacity of cryopreserved tissue is of primary importance. This study provides the first quantitative evidence of the impact of alkylating agents on *in vitro* viability and developmental capacity of ovarian tissue that was cryopreserved for fertility preservation. We demonstrated that previous chemotherapy significantly impaired the survival and development of the ovarian follicles in culture. The cyclophosphamide equivalent dose was an independent predictor for the density of atretic follicles. The study demonstrates that the youngest patients, exposed to the highest cumulative doses of alkylating agents, had the highest probability of increased follicular atresia and the lowest probability of developing follicles after culture.

Previous analyses of clinical histological samples and the results of xenotransplantation studies show that chemotherapy, in particular with alkylating agents, induces atresia of ovarian follicles [29–31]. Patients who received chemotherapy presented with significantly lower primordial follicle counts in morphological analysis and decreased estrogen production *in vitro* [32]. Cyclophosphamide is one of the most widely used alkylating agents. It is known to have an adverse effect on rapidly dividing cells and it damages DNA repair mechanisms [33]. In the ovary, granulosa cells surrounding the oocytes are an important target of alkylating agents [15;34]. DNA damage can lead to apoptosis of proliferating granulosa cells and decrease intercellular communication between them and the oocyte [35]. Alkylating agents may also directly cause DNA and RNA damage and therefore affect even non-dividing ovarian follicles [35;36]. It has also been suggested that ovarian exposure to cyclophosphamide may trigger dormant follicle activation, resulting in burnout of the follicular reserve [15;37].

As pre-pubertal girls have more primordial follicles than adults, it has been assumed that their follicle cohort has a higher maturation potential and better survival capacity after cancer treatment [38;39]. However, this hypothesis has been questioned after a recent *in vitro* study suggesting that there may be key differences in follicular recruitment and development between the ovaries of young girls and adults. The capacity of primordial follicles to be activated and reach the secondary stage of development may be age-dependent [40]. The present results confirm decreased *in vitro* viability and development of ovarian follicles from very young girls. Increased age correlated with fewer follicles, more developing and fewer atretic follicles after culture. A study on mouse ovaries revealed two classes of primordial follicles; the first wave, which are activated immediately after they are formed, and adult primordial follicles, which are activated gradually during reproductive life [41]. These two primordial follicle pools have been shown to differ in their developmental dynamics and location in the maturing ovary [42]. Our results may indicate that a similar situation exists in humans. A decreased rate of activation and development of follicles in ovaries from young girls may reflect compromised developmental competence *in vitro*. Presently no international consensus exists on the age at which reproductive potential is actually reached making it unclear how recommendations for fertility preservation can be effectively applied to cancer patients younger than 18 years old [1].

The present study population was small and heterogeneous with a significant difference in the median age of patients who underwent chemotherapy versus those who did not. Ideally, the effect of chemotherapy should have been studied in groups of comparable age and compared to age-matched control tissue. This was not possible because of the clinical nature of the samples available. The fact that first-line therapy seldom associates with subfertility among pre-pubertal girls delays the decision of fertility preservation. Childhood cancers also require prompt initiation of cancer therapy often before ovarian biopsy. For ethical reasons, it was not possible to collect age-matched tissue from healthy young girls. The healthy control ovarian tissue cultures were from few adult samples. Their performance in *in vitro* conditions was comparable with those in previous [6;13;25]. In order to decrease the effect of skewed study populations, the entire study material was included in multivariate analysis to adjust for differences in age, use of cryoprotectant agents and exposure to CED. The analysis identified CED and age as independent predictors of follicle survival. No independent effect on viability of the follicles was found to be associated with the use of either PrOH or EG as cryoprotectants during slow freezing. This is in agreement with the results of previous studies [26;43].

In conclusion, the results of this study demonstrate that exposure to an increased cumulative dose of alkylating agents prior to ovarian cryopreservation decreases survival of cultured human ovarian follicles. Therefore, if possible, fertility preservation should be carried out before initiation of chemotherapy. The findings further confirm that the capacity of ovarian follicles to survive and develop in culture may be reduced among young girls. The results of the present study can have implications for future implementation, timing and quality control of fertility-preservation methods.
